# Chlorogenic acid attenuates tet (X)-mediated doxycycline resistance of *Riemerella anatipestifer*

**DOI:** 10.3389/fvets.2024.1368579

**Published:** 2024-05-03

**Authors:** Yuwen Han, Min Li, Dehai Su, Shiyu Xiong, Youshu Feng, Qin Deng, Huanzhong Ding

**Affiliations:** Guangdong Key Laboratory for Veterinary Drug Development and Safety Evaluation, College of Veterinary Medicine, South China Agricultural University, Guangzhou, China

**Keywords:** *Riemerella anatipestifer*, resistance, tet (X), doxycycline, chlorogenic acid

## Abstract

**Introduction:**

The increasing resistance of *R. anatipestifer* has posed a significant threat to the poultry industry in recent years. The tet gene is the primary determinant of tetracycline resistance in numerous bacteria, and the enzyme modification gene tet(X) is predominantly detected in tetracycline-resistant *R. anatipestifer* strains.

**Methods:**

In this study, we evaluated the susceptibility of both the standard strain and clinical isolates of *R. anatipestifer* to doxycycline. And the expression levels of tet(X), tet(A), and tet(O) genes were detected. To assess drug susceptibility, shuttle plasmids were constructed to transfer the tet(X) gene into the standard strain of *R. anatipestifer* followed by treatment with chlorogenic acid.

**Results and discussion:**

The results revealed that the minimum inhibitory concentration of doxycycline for the standard strain was 0.25μg/mL, whereas it exceeded 8μg/mL for the clinical isolates. Furthermore, there was a significant upregulation observed in expression levels of tet(X), tet(A), and tet(O) genes among induced strains. Interestingly, when transferring the tet(X) gene into the standard strain, its sensitivity to doxycycline decreased; however, MIC values for chlorogenic acid remained consistent between both standard and drug-resistant strains of *R. anatipestifer*. Moreover, we made a surprising discovery that screening passage with chlorogenic acid resulted in increased sensitivity of *R. anatipestifer* to doxycycline. Further analysis demonstrated a reversal in expression trends among three differentially expressed genes within induced drug resistance group after intervention with chlorogenic acid. The main objective behind this study is to investigate both killing effect exerted by chlorogenic acid on drug-resistant *R. anatipestifer* as well as its regulatory impact on drug resistance genes. This will provide novel insights and theoretical basis towards development of chlorogenic acid as a promising drug for treatment and control of drug resistance in *R. anatipestifer*.

## 1 Introduction

*Riemerella anatipestifer* (*R. anatipestifer*) is a bird pathogen found all over the world. It mainly infects ducks, geese, and turkeys and causes characteristic serositis and septicemia (1). However, feeding management cannot keep up with the development speed of intensive breeding, which leads to increasingly serious secondary infections of *R. anatipestifer* (2). Because *R. anatipestifer* is prone to drug resistance and the irrational use of a large number of antibiotics in clinics, the drug resistance of *R. anatipestifer* to antibiotics is increasing year by year. At present, doxycycline, a tetracycline antibiotic, is an important antibacterial drug to prevent or treat *R. anatipestifer* infection (2). Tetracycline antibiotics are cheap broad-spectrum antibiotics that are commonly used in clinics. They have a strong antibacterial effect on Gram-negative bacteria (3, 4). There are a large number of tetracycline-resistance determinants in *R. anatipestifer*, which make them naturally resistant to tetracycline, such as tet (M), tet (A), tet (O), tet (Q), and tet (B) (5–7). *R. anatipestifer* is generally resistant to tetracycline antibiotics. The indiscriminate use of tetracycline has led to the widespread of drug-resistance genes. The Tet gene is the main reason for the resistance of many bacteria to tetracycline drugs (8, 9). The enzyme-modified gene tet (X) was detected most in tetracycline-resistant strains, and more than 80% of tetracycline-resistant strains contained tet (X) (10, 11). In addition, tet (A) and tet (B) genes of the tetracycline efflux pump and tet (M), tet (O), and tet (Q) genes of ribosomal protective protein synthesis in *R. anatipestifer* were reported for the first time (10). In 2019, *R. anatipestifer* disease broke out on a duck farm in Guizhou Province, and doxycycline treatment was ineffective. The drug sensitivity test of a strain of *R. anatipestifer* isolated showed that it was resistant to tetracycline and doxycycline, and the tetracycline-resistance gene tet (B) was detected, which made the bacteria resistant to antibiotics by encoding the efflux pump (12).

In recent years, with the continuous emergence of drug-resistant strains, the use of tetracycline drugs has been limited. Traditional Chinese medicine has played an active role in modern prevention and the control of bacterial infections (13, 14). Some traditional Chinese medicine has high antibacterial activity and is not easy to induce bacterial resistance (15, 16). Therefore, the research and development of antibacterial traditional Chinese medicine is of great significance in solving the problem of the generation of drug-resistant strains and the shortage of antibiotics. There are lots of traditional Chinese medicine resources in China. Through research studies, it has been reported that a variety of active ingredients in traditional Chinese medicine have good inhibitory effects on drug-resistant bacteria and can even eliminate or reverse the drug resistance of bacteria (17–19). This study mainly explored the killing effect of chlorogenic acid on drug-resistant *R. anatipestifer* and its regulation of drug-resistant genes, which will provide new ideas and a theoretical basis for the development of chlorogenic acid as a new drug for the treatment and control of *R. anatipestifer* resistance.

## 2 Materials and methods

### 2.1 Strains

*Riemerella anatipestifer* (ATCC11845) was purchased from the China Veterinary Drug Administration. The clinical strains were isolated from the diseased materials of various duck farms in Guangzhou and were identified and preserved by the Veterinary Pharmacology Laboratory of South China Agricultural University.

### 2.2 Culture of *Riemerella anatipestifer*

A small amount of bacterial solution was dipped in the glycerin meat solution suspension using an inoculation ring, scribed, and inoculated on the tryptone soy agar (TSA) [Qingdao Hope Bio-technology Co., Ltd.] plate containing 5% fetal bovine serum and placed on the incubator containing 5% CO_2_ at 37°C for 24 h in sterile conditions. A single colony can be used for further tests. The single colony was isolated and inoculated in Tryptic-Soytone-Broth-Medium (TSB) [Qingdao Hope Bio-technology Co., Ltd.] medium containing 5% fetal bovine serum and cultured for 10 h to complete the logarithmic period for the test.

### 2.3 The determination of minimum inhibitory concentration (MIC)

The MIC of doxycycline and chlorogenic acid against *R. anatipestifer* was determined by broth microdilution method according to the regulations of the (20). The drug stock solution was thawed at room temperature and thawed using 0.22 μm sterile membrane sterilization, diluted to the appropriate concentration with blank TSB medium. In a 96-hole plate, 100 μL of blank medium was added into 1–9 holes, and then 100 μL of diluted drug was added in the first hole. After mixing the blank medium and the diluted drug with a multi-channel pipette in the first hole, take 100 μL from the first hole add into the second hole,dilute to the ninth hole by double dilution, and discard 100 μL in the ninth hole. The final concentration range of doxycycline was 0.0625–16 μg/mL. The final concentration range of chlorogenic acid was 0.25–64 mg/mL. Then the bacterial suspension in the logarithmic phase was taken, diluted to 10^6^ CFU/mL, and 100 μL of bacterial solution was added into 1–9 wells, respectively, so that the final bacterial concentration is 5 × 10^5^ CFU/mL. Now, 100 μL of bacterial liquid and 100 μL of blank broth were added into hole 11, which acts as a positive control. Then, 200 μL of blank broth was added into hole 12, which was used as a negative control. The plate was put into the incubator containing 5% CO_2_ at 37°C for 18 h and then taken out to observe the results. The minimum drug concentration that can inhibit the growth of bacteria as observed by the naked eye is recorded as the MIC of doxycycline or chlorogenic acid on *R. anatipestifer*. Three parallels were set, and the operation was repeated three times.

### 2.4 Single antibiotic-induced resistant *Riemerella anatipestifer*

*Riemerella anatipestifer* was induced continuously with doxycycline of different concentrations. A total of 1–3 passages were co-cultured with doxycycline of 1/2MIC concentration, 4–5 passages were co-cultured with doxycycline of 1MIC concentration, 6–8 passages were co-cultured with doxycycline of 2MIC concentration, and 9–10 passages were co-cultured with doxycycline of 4MIC concentration. The single induction time was 12 h.

### 2.5 The detection of gene differences among different strains of *Riemerella anatipestifer* by transcriptomics

The standard strain (ATCC11845) and the induced strain were scraped from the plate into the TSB liquid medium, and the OD was adjusted to 1 and cultured in the TSB liquid medium for 2 h, and the bacteria were collected. The total RNA was extracted using the bacterial total RNA extraction kit [Tiangen Biochemical Technology (Beijing) Co., Ltd., China], and the RNA purity was determined using NanoDrop 2000, and then sent to Lianchuan Biological Company for quality inspection. After passing the quality inspection, the transcriptome was detected. The raw data of transcriptome sequencing were filtered to obtain high-quality data information; DESeq v1.20.0 software was used to study the differentially expressed genes among the three groups. The conditions were set as the difference multiple |log_2_FC| > 1, with a significance *p*-value of <0.05. The mRNA of the three samples was screened for differentially expressed genes.

### 2.6 The detection of tetracycline-resistance gene content in standard strains and induced resistant strains

In order to verify the reliability of transcriptome data, the RNA of the standard strain (ATCC11845) and induced strain were extracted according to the same transcriptome sampling method (see 2.5). The RNA samples were subjected to a 20 μL system that was reverse transcribed into cDNA. The tetracycline-resistance genes tet (X), tet (O), and tet (A), which were characterized by standard strain (ATCC11845) and induced strain, were detected by RT-PCR.

### 2.7 Mic changes of doxycycline in *Riemerella anatipestifer* after standard bacteria were transferred into drug-resistance gene

Using the primer sets tet (X), tet (O), and tet (A) listed in Table 1, the tet (X), tet (O), and tet (A) genes were amplified from the *R. anatipestifer* induced strain by PCR. The purified amplicons were sequenced and digested with restriction enzymes and then connected to the same digestion shuttle vector pLMF03 [Ordered by Miaoling Biotechnology Co., Ltd.]. Then the corrected recombinant plasmid was introduced into *E. coli* S17-1 [Miaoling Biotechnology Co., Ltd.]. These sequences were analyzed by BLAST in NCBI.

**Table 1:** Primers used in RT-PCR.

Gene	Primer (5′ → 3′)
tet (A)	Forward: GCTACATCCTGCTTGCCTTC
	Reverse: CATAGATCGCCGTGAAGAGG
tet (O)	Forward: AACTTAGGCATTCTGGCTCAC
	Reverse: TCCCACTGTTCCATATCGTCA
tet (X)	Forward: ATGCAAATGCGAATAGATACAGAC
	Reverse: CAATTGCTGAAACGTAAAGTC

As mentioned above, the correct recombinant plasmid was transferred to *R. anatipestifer* (ATCC 11845) by coupling transfer (21, 22). In short, *Escherichia coli* S17-1 containing the recombinant plasmid was used as the donor strain, while the reference strain ATCC 11845, which does not carry any tet gene and is sensitive to tetracycline, was used as the recipient strain (23). Meanwhile, the negative control empty vector pLMF03 was transferred to ATCC 11845 to obtain the transconjugant ATCC 11845 [pLMF03]. The MIC of doxycycline of the transconjugant was measured as described above.

### 2.8 Mic changes of doxycycline in induced strain *Riemerella anatipestifer* after the selective passage of chlorogenic acid

The induced strain, *R. anatipestifer,* was continuously cultured with 1/2MIC chlorogenic acid for 12 h. After each generation of culture, the MIC of doxycycline was detected.

### 2.9 Changes in resistance genes of *Riemerella anatipestifer* after the action of chlorogenic acid

The induced strain *R. anatipestifer* was continuously cultured with 1/2MIC chlorogenic acid for 12 h. After each generation of culture, the transcription level of the tetracycline-resistance gene tet (X) was detected.

### 2.10 The effect of chlorogenic acid on the drug resistance of *Riemerella anatipestifer* in sheldrake

The sheldrakes utilized in this experiment were procured from the Guangxi Guiliu Poultry Nanning Hatching Branch, and the experiment has received approval from the Ethics Committee of Experimental Animals at South China Agricultural University (No. 2022A022). In total, 60 3-day-old male sheldrakes were randomly allocated into three groups, with 20 ducks per group. Following 5 days of acclimation feeding, each duck was intraperitoneally administered with 1 mL of solution containing 10^9^ CFU/mL of *R. anatipestifer* (induced resistant *R. anatipestifer* in 2.4). The doxycycline group received a dose of 20 mg/kg doxycycline via intramuscular injection to the thigh region, 12 h post-infection; the chlorogenic acid group was orally administered with a dose of 8 g/kg of chlorogenic acid by gavage, also 12 h after infection, while the control group received an equivalent volume of dilution solution (glyceraldehyde:normal saline =40:60, V/V) through intramuscular injection to the thigh region, 12 h post-infection. The drug administration occurred every 12 h for 10 consecutive doses.

All sheldrakes in the above groups were euthanized after 24 h of drug administration, and 4 ducks were randomly euthanized every 24 h. After euthanizing the ducks, the heart tissue was collected aseptically, and 1 g of the heart tissue was accurately weighed and added to 1 mL of normal saline for homogenization using a handheld homogenizer. The heart tissue was diluted 10 times to an appropriate gradient, and 20 μL of each series of diluent was dropped on the TSA plate containing 5% bovine serum. The strains were incubated at 37°C in a 5% CO_2_ incubator for 20–24 h, and a single colony was selected for culture. The MIC of doxycycline and the expression of the tet (X) gene of *R. anatipestifer* were detected after different administration times.

### 2.11 Data analysis

GraphPad prism 9.4.1 software was used for data processing and plotting, and a *t*-test was used for statistical analysis. When the analysis result was a *p*-value of <0.05, there was a statistical difference.

## 3 Results

### 3.1 The MIC of doxycycline and chlorogenic acid on *Riemerella anatipestifer*

The MIC of doxycycline and chlorogenic acid against *R. anatipestifer* was determined by the broth microdilution method according to the regulations of the CLSI (2013). The results showed that the MIC of doxycycline in the standard strain (ATCC11845) was 0.25 μg/mL. The MIC of chlorogenic acid in the standard strain (ATCC11845) was 4 mg/mL. The MIC results of clinical isolates are shown in Table 2.

**Table 2:** The MIC results of clinical isolates.

Strain number	Doxycycline (μg/ml)
ATCC 11845	0.25
LCFL-01	32
LCFL-02	128
LCFL-03	8
LCFL-04	8
LCFL-05	32
LCFL-06	16
LCFL-07	8
LCFL-08	128
LCFL-09	32

### 3.2 Induction and MIC detection of resistant *Riemerella anatipestifer*

After 10 generations of induction, we obtained a new drug-resistant strain, and the MIC of doxycycline to the induced drug-resistant strain increased from 0.25 μg/mL to 2 μg/mL. It is surprising to note that the MIC of chlorogenic acid to the induced drug-resistant strains did not change and was still 4 mg/mL.

### 3.3 The quality assessment of sequencing data

In the established sequencing library, the proportion of base quality exceeding Q20 in the three groups of samples was more than 94.20%, and the proportion exceeding Q30 was more than 94.14% (Table 3), indicating that the sequencing results were good.

**Table 3:** Quality assessment of sequencing data.

Term	RA_clinical	RA_induce	RA_standard
TOTAL_READS	10,189,342 (100.00%)	10,184,261 (100.00%)	10,173,024 (100.00%)
MAPPED_READS	9,894,276 (97.11%)	9,886,881 (97.08%)	9,871,414 (97.04%)
TARGET_TERRITORY	2,175,511	2,175,511	2,175,511
MEAN_TARGET_COVERAGE	601.40	601.21	600.61
PCT_TARGET_BASES_30X	94.14%	94.66%	95.25%
PCT_TARGET_BASES_20X	94.20%	94.75%	95.31%
PCT_TARGET_BASES_10X	94.30%	94.81%	95.39%
PCT_TARGET_BASES_2X	94.51%	94.93%	95.55%
PCT_TARGET_BASES_1X	94.62%	95.02%	95.71%

### 3.4 The analysis of differentially expressed genes

**Figure 1:**
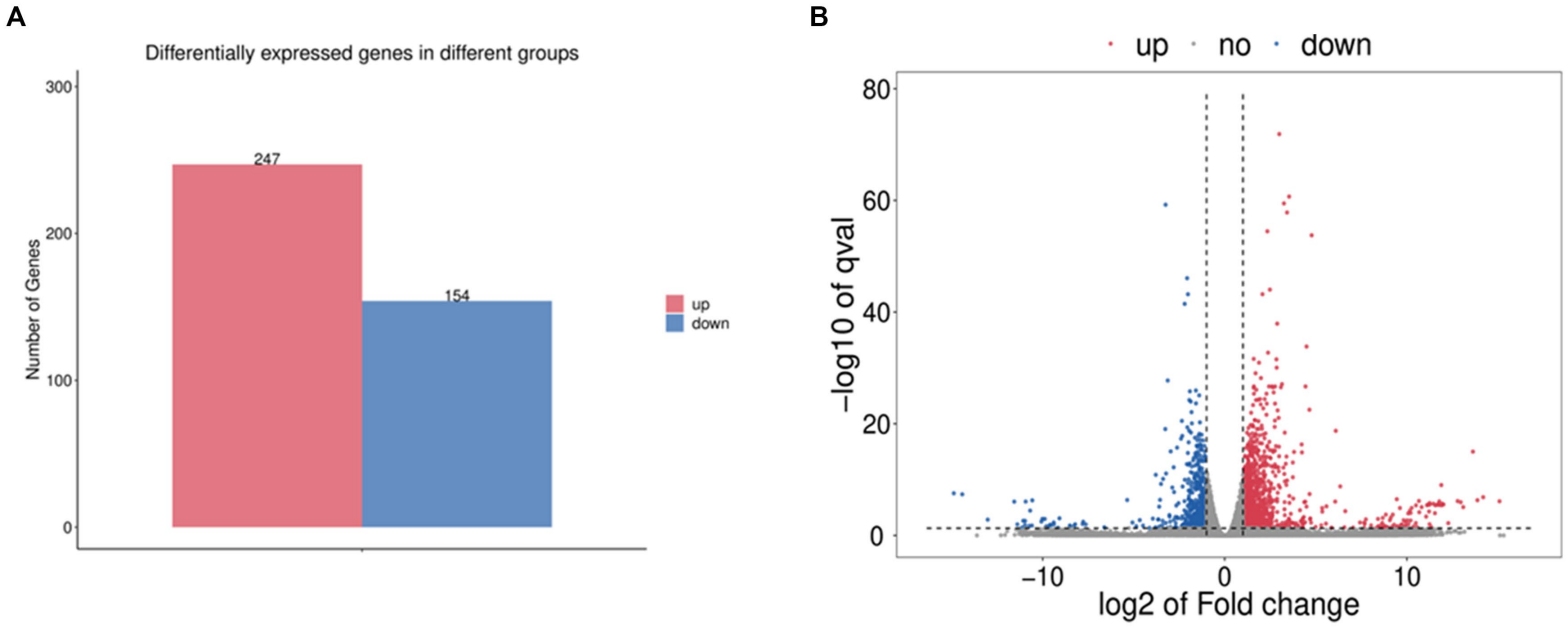
**(A)** Statistical histogram of differential genes. **(B)** Differential gene volcano map. Among them, red represents upregulated significantly differentially expressed genes, blue represents downregulated significantly differentially expressed genes, and gray dots represent non-significantly differentially expressed genes.

The gene data obtained by RNA SEQ were analyzed for differential expression using DESeq v1.20.0 software and screened under the conditions of |log_2_FC| > 1, *p* < 0.05. The results showed that compared with the standard strain, there were 341 differentially expressed genes in the induced drug-resistant strain, of which 247 were upregulated and 154 were downregulated (Figure 1).

**Figure 2:**
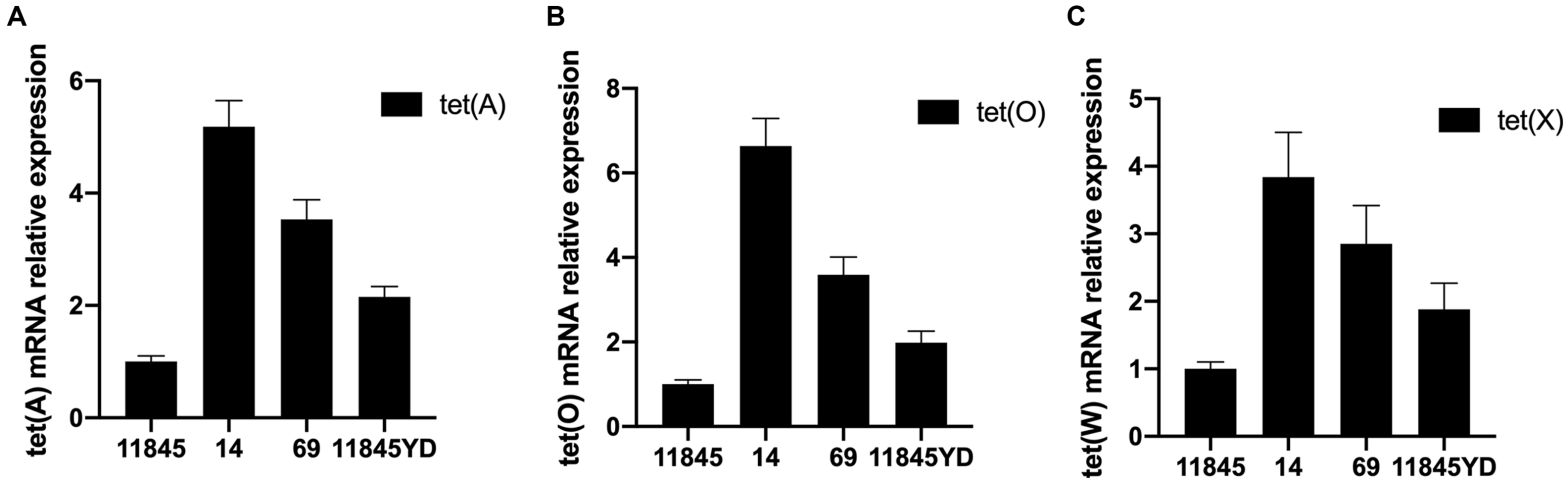
The expression of drug-resistance genes detected by RT-PCR. **(A)** Expression of tet (A). **(B)** Expression of tet (O). **(C)** Expression of tet (X).

The results of RT-PCR showed that the content of drug-resistance genes of antibiotic-induced resistant strains was significantly higher than that of standard strains. The content of drug-resistance genes of clinical isolates was also significantly higher than that of standard strains. With the increase in MIC value, the content of drug-resistance genes also increased. The results are shown in Figure 2. Therefore, it can be preliminarily concluded that the resistance of *R. anatipestifer* to tetracycline antibiotics may be related to tet (A), tet (O), and tet (X) genes.

### 3.5 Improvement of the MIC of doxycycline on *Riemerella anatipestifer* after standard bacteria were transferred into drug-resistance gene

**Figure 3:**
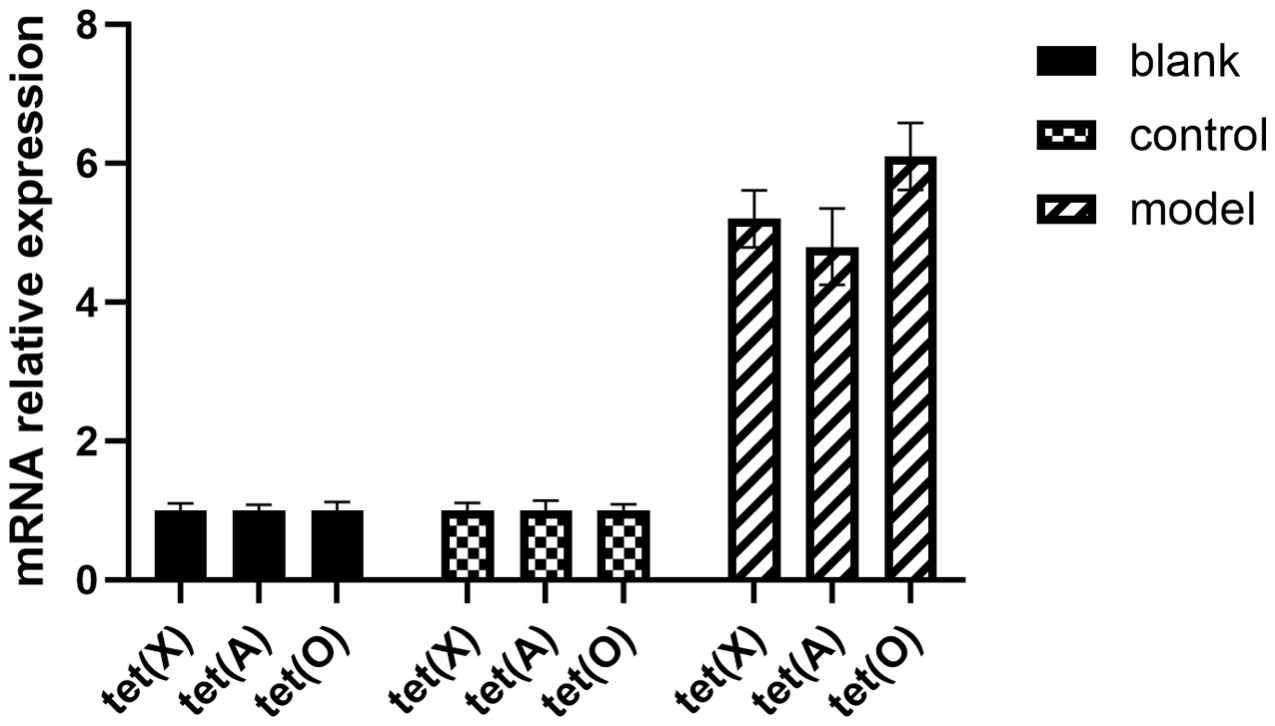
The expression levels of drug-resistance genes tet (A), tet (O), and tet (X) in different groups after standard bacteria were transferred into drug-resistance gene.

*E. coli* S17-1 pLMF03:: tet (X), *E. coli* S17-1 pLMF03:: tet (A), and *E. coli* S17-1 pLMF03:: tet (O) were used as the donor bacteria. RA ATCC 11845 was used as the recipient bacteria, the donor bacteria was mixed with the recipient bacteria at 1:4, and coated on the filter membrane. After being cultured on the blood plate, the bacteria were washed with magnesium chloride and screened on the blood plate containing kanamycin and cefoxitin resistance. The results as shown in Figure 3, tet (X), tet (A), and tet (O) fragments could be amplified by PCR, indicating that *R. anatipestifer* ATCC 11845 pLMF03:: tet (X), *R. anatipestifer* ATCC 11845 pLMF03:: tet (A), and *R. anatipestifer* ATCC 11845 pLMF03:: tet (O) transgenic strains were successfully constructed. The MIC of doxycycline on *R. anatipestifer* ATCC 11845 pLMF03:: tet (X), *R. anatipestifer* ATCC 11845 pLMF03:: tet (A), and *R. anatipestifer* ATCC 11845 pLMF03:: tet (O) transgenic strain was determined by the broth microdilution method. The results showed that the MIC of doxycycline on *R. anatipestifer* ATCC 11845 pLMF03:: tet (X) transgenic strain increased to 4 μg/mL, the MIC of doxycycline on *R. anatipestifer* ATCC 11845 pLMF03:: tet (A) transgenic strain increased to 1 μg/mL, and the MIC of doxycycline on *R. anatipestifer* ATCC 11845 pLMF03:: tet (O) transgenic strain increased to 2 μg/mL. However, the MIC of the negative control empty vector group was still 0.25 μg/mL. The results are shown in Figure 3 and Table 4.

**Table 4:** MIC of doxycycline on *R. anatipestifer* after standard bacteria were transferred into drug-resistance gene.

Transferred gene	Doxycycline (μg/ml)
tet (X)	4
tet (A)	1
tet (O)	2
Control	0.25

### 3.6 The sensitivity of doxycycline to induced strain *Riemerella anatipestifer* increased after the selective passage of traditional Chinese medicine

The chlorogenic acid concentration of 1/2MIC was selected to act on the induced drug-resistant strains. After continuous passage, the MIC of doxycycline was detected again. We were surprised to find that the sensitivity of *R. anatipestifer* to doxycycline was improved after the selective passage of chlorogenic acid. Specific MIC values are shown in Table 5.

**Table 5:** MIC of doxycycline to induced strain *R. anatipestifer*.

Cultivation times	Doxycycline (μg/ml)
0	2
1	2
2	2
3	2
4	1
5	1
6	1
7	1
8	0.5
9	0.5

### 3.7 The expression of drug-resistance genes in *Riemerella anatipestifer* decreased with the passage of traditional Chinese medicine

**Figure 4:**
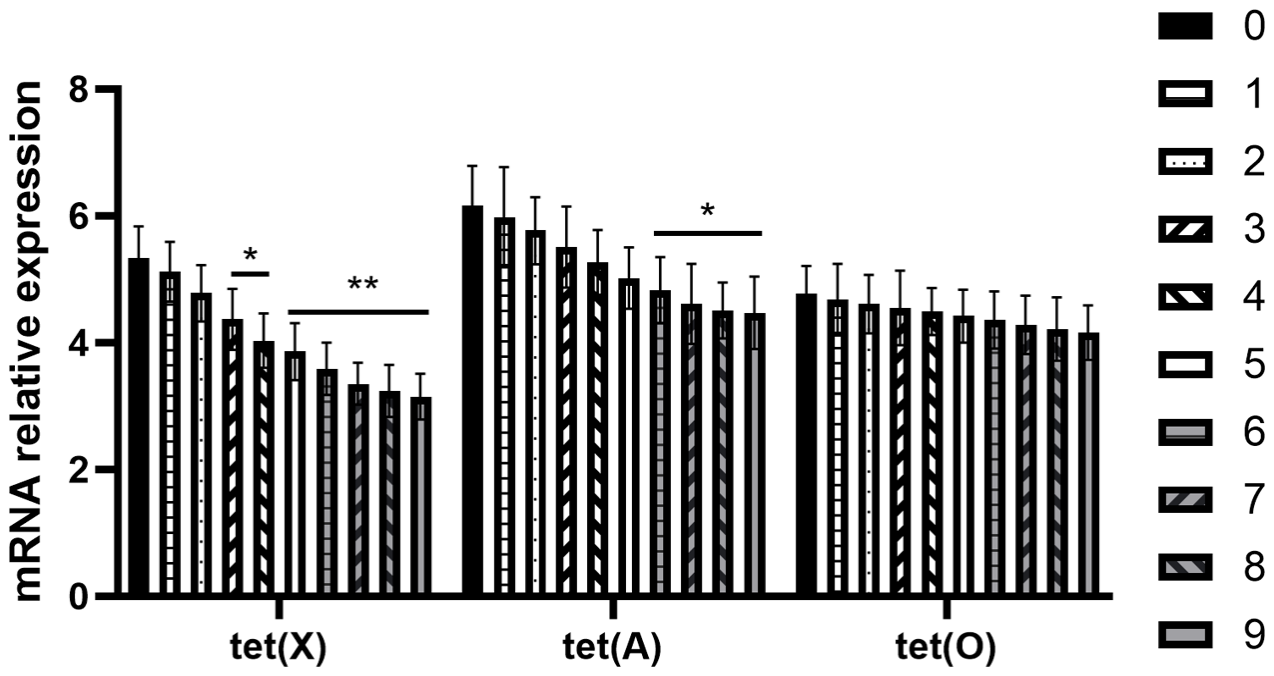
Changes in resistance genes of *R. anatipestifer* after exposure to chlorogenic acid. Compared with the “0” group, ∗*p* < 0.05, ∗∗*p* < 0.01.

The expression of *R. anatipestifer*-resistance genes is shown in Figure 4. It can be seen from the above results that the expression trend of three differential genes, namely tet (A), tet (O), and tet (X) genes, in the induced drug-resistance group was reversed after the chlorogenic acid intervention, and the downregulation level of tet (X) gene was found to be the most significant.

### 3.8 The effect of chlorogenic acid on the drug resistance of *Riemerella anatipestifer* in sheldrake

**Figure 5:**
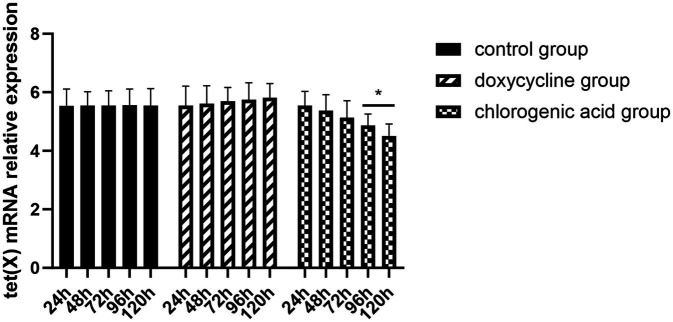
Changes in the tet (X) gene of *R. anatipestifer* after exposure to chlorogenic acid. Compared with the “24 h” each group, ∗*p* < 0.05, ∗∗*p* < 0.0.

The results demonstrated that the MIC of the control group, the doxycycline group, and the chlorogenic acid group remained at 2 μg/mL after 24 h, 48 h, and 72 h of administration. At 96 h and 120 h post-administration, the MIC of the control and doxycycline groups remained at 2 μg/mL, while the chlorogenic acid group exhibited a reduced MIC of 1 μg/mL. Figure 5 illustrates the expression of the tet (X) gene. It is evident that there was minimal variation in the tet (X) gene expression within the control group across different time points. In contrast, a slight increase in the tet (X) gene expression was observed in the doxycycline group with prolonged drug administration time. Conversely, a decrease in the tet (X) gene expression was noted in the chlorogenic acid group as drug administration time increased.

## 4 Discussion

Among ducks infected with *R. anatipestifer*, drug treatment is the most common approach. Tetracyclines have been widely used for disease management and growth promotion in livestock (4, 24). However, both clinical practice and laboratory research have revealed that *R. anatipestifer* exhibits resistance to a variety of antibiotics, with its resistance spectrum showing an increasingly broad trend (25). Our experiments also confirmed that *R. anatipestifer* clinical isolates were resistant to doxycycline. Although it has been found that *R. anatipestifer* is resistant to antibiotics such as chloramphenicol, penicillin, kanamycin, gentamicin, lincomycin, and florfenicol (12), the majority of the resistance genes and mechanisms in *R. anatipestifer* remain unknown.

The genotypes of tetracycline resistance were found to be highly prevalent in *R. anatipestifer* isolates, encompassing efflux genes [tet (A) and tet (B)], ribosomal protection genes [tet (M), tet (O), and tet (Q)], enzymatic gene tet (X), and mosaic tetracycline-resistance gene tet (O/W/32/O), as confirmed by De-Kang Zhu et al. (10). The overall rate of positive-resistance genes was 90.6%, which was remarkably high. In our study, we observed a significant upregulation of the expression levels of tet (X), tet (A), and tet (O) genes in both inducible resistant strains and clinical isolates compared to standard strains. These findings suggest a potential association between alterations in the expression of tet (X), tet (A), and tet (O) genes and tetracycline resistance in *R. anatipestifer*.

Tetracycline antibiotics do not confer active advantages in organisms, primarily due to the enzymatic degradation; however, the precise spatial structure of tetracycline-inactivating enzymes remains poorly understood. The tet (X) gene represents a novel drug-resistance gene capable of directly inactivating tetracycline (26). The emergence and dissemination of plasmids carrying the tet (X)-resistance gene and its variants have resulted in reduced efficacy of tetracycline antibiotics (27). In this study, we initially constructed a standard strain harboring the tet (X) gene and compared its susceptibility to doxycycline with that of wild-type strains. Our findings revealed decreased sensitivity of the tet (X) gene standard strain toward doxycycline, while no change was observed in the sensitivity of the negative control vector group. These results highlight the significance of the resistance gene tet (X) as a key factor contributing to diminished drug susceptibility among resistant strains.

In this study, we observed that the MIC of chlorogenic acid against both the standard strain and the tetracycline-resistant strain of *R. anatipestifer* was identical. Moreover, it was pleasantly surprising to discover that the sensitivity of *R. anatipestifer* to doxycycline improved following selective exposure to chlorogenic acid. Further analysis revealed a reversal in the expression pattern of three differentially regulated genes, namely the tet (A), tet (O), and tet (X) genes, in the induced resistance group after intervention with chlorogenic acid. This suggests that the regulatory mechanism underlying chlorogenic acid’s resistance against tetracycline-resistant *R. anatipestifer* is associated with modulating the expression of these genes. Notably, there was a significant downregulation observed in the tet (X) gene, indicating its potential role as a primary target for restoring sensitivity to doxycycline in resistant *R. anatipestifer* induced by chlorogenic acid treatment. The identical outcomes were observed *in vivo*. The tet (X) gene represents a novel drug-resistance gene capable of directly deactivating tetracycline. Overall, our findings demonstrate that chlorogenic acid can reverse the increasing trend of drug-resistant strains carrying tet (X), significantly reducing its expression level (*p* < 0.05). This suggests that suppressing the expression of the drug-resistant gene tet (X) may be one crucial mechanism through which chlorogenic acid mitigates resistance in *R. anatipestifer*.

The flavin-dependent monooxygenase tet (X), consisting of 388 amino acids, relies on NADPH, Mg^2+^, and O_2_ for the monohydroxylation at the C-11a position. Subsequently, it undergoes intramolecular cyclization and non-enzymatic decomposition to generate other metabolites. This enzyme not only catalyzes the degradation of the first- and second-generation tetracycline antibiotics but also exhibits catalytic activity toward the third-generation antibiotic tigecycline (28). Considering that tet (X) modification of tetracycline antibiotics is dependent on reactive oxygen species, previous studies have demonstrated that chlorogenic acid can mitigate oxidative damage and reduce reactive oxygen species production (29–31). Therefore, it is speculated that chlorogenic acid’s potential reduction in *R. anatipestifer*’s sensitivity to doxycycline may be attributed to its remarkable antioxidant function, which requires further verification.

## Data availability statement

The data presented in the study are deposited in the NCBI SRA repository, accession number are SRR28639081, SRR28639082, SRR28639083, SRR28639078, SRR28639079, SRR28639080.

## Author contributions

YH: Writing – review & editing, Writing – original draft. ML: Writing – original draft. DS: Writing – original draft. SX: Writing – original draft. YF: Writing – original draft. QD: Writing – original draft. HD: Writing – review & editing.
